# A novel targeted hybrid capture-NGS assay for sensitive detection of multiplex respiratory pathogens

**DOI:** 10.1128/spectrum.02908-25

**Published:** 2025-11-17

**Authors:** Junyan Ma, Liping Yu, Kangchen Zhao, Qiao Qiao, Xiaojuan Zhu, Tao Wu, Heng Rong, Shuo Ning, Jinlei Guo, Yuhan Ding, Ying Chi, Lunbiao Cui, Yiyue Ge

**Affiliations:** 1 NHC Key Laboratory of Enteric Pathogenic Microbiology, Jiangsu Provincial Medical Key Laboratory of Pathogenic Microbiology in Emerging Major Infectious Diseases, Jiangsu Provincial Center for Disease Control and Prevention 12666, Nanjing, China; 2 School of Public Health, Nanjing Medical University 12461, Nanjing, China; Children's National Hospital, Washington, DC, USA

**Keywords:** hybrid capture, MT-capture, tNGS, respiratory pathogens, multiplex detection

## Abstract

Emerging respiratory infectious diseases represented by COVID-19, along with traditional respiratory infections, pose a serious threat to human health. High-throughput sequencing (NGS), with its high sensitivity and ultra-high throughput, is particularly suitable for the detection of respiratory pathogens (RP) that are extremely diverse in types and frequently involved in mixed infections. In this study, by integrating a Micro-Targets Hybrid Capture (MT-Capture) system, we developed previously with NGS, we developed a novel assay (termed RP-MT-Capture NGS) for the detection of multiple respiratory pathogens (more than 300 species/types). By optimizing probe design and hybridization capture procedures, RP-MT-Capture NGS achieved high detection sensitivity for different types of pathogens. For influenza viruses, this assay could acquire full-length sequences of hemagglutinin (HA) and neuraminidase (NA) genes for samples with CT values < 32, offering a robust tool for viral mutation surveillance and recombination analysis. The results of clinical sample detection showed that RP-MT-Capture NGS exhibited superior accuracy and sensitivity compared to TaqMan array and metagenomic NGS (mNGS) technologies for respiratory pathogen detection. Compared with traditional probe hybridization-based targeted NGS (tNGS), RP-MT-Capture NGS significantly shortens the wet lab experiment time to within 6 h. In summary, the RP-MT-Capture NGS assay developed in this study offers a novel tool for detecting multiple respiratory pathogens, with substantial clinical and public health relevance.

**IMPORTANCE:**

Emerging and traditional respiratory infections pose threats to human health. These diseases are caused by a variety of pathogens, which often lead to co-infections and, thus, make detection difficult. This study combines a novel probe hybridization capture system with high-throughput sequencing to develop a new detection tool (RP-MT-Capture NGS), which can identify over 300 types of respiratory pathogens. For influenza viruses, it can reveal complete details of key viral genes, facilitating the tracking of viral mutations. Compared with existing detection methods, this new tool is more accurate, more sensitive, and has a higher throughput. It provides great value for clinical practice and public health in respiratory pathogen detection.

## INTRODUCTION

Respiratory infectious diseases have long been a global public health focus due to their high incidence and mortality (1, 2). In recent years, globalization has driven the emergence of multiple novel respiratory infectious diseases with epidemic potential. Examples include severe acute respiratory syndrome (SARS), Middle East respiratory syndrome (MERS), human infections with highly pathogenic avian influenza (H5N1, H7N9), and Coronavirus Disease 2019 (COVID-19) (3, 4). Key contributing factors include rapid population mobility, antibiotic misuse, environmental pollution, and pathogen mutation and evolution. Besides emerging diseases, traditional respiratory pathogens also frequently cause infections and epidemics. Particularly in the post-COVID-19 era, the population’s “immune debt (a phenomenon where the overall population’s immune protection against certain pathogens decreases due to reduced exposure, potentially leading to increased infection rates when exposure resumes)” has led to a substantial increase in the incidence of common respiratory infectious diseases (5–7). Notably, the two most devastating infectious disease pandemics of the past century, the 1918 Spanish flu and the 21st-century COVID-19 outbreak, were both triggered by respiratory infections. The short incubation period, high contagiousness, and broad susceptibility of the population to respiratory pathogens pose significant challenges to the prevention and control of respiratory infectious diseases (8). Rapid, comprehensive, and accurate pathogen diagnosis is the cornerstone of treating and preventing respiratory infectious diseases, playing a crucial role in the early selection of sensitive medications and timely implementation of effective prevention and control measures.

Conventional laboratory diagnostic approaches, including pathogen culture, biochemical assays, and immunological detection, are inherently time-consuming, labor-intensive, and limited in sensitivity (9–11). Furthermore, the “window period (the time interval between the initial infection with a pathogen and the point when the infection can be reliably detected by current diagnostic methods)” of infectious diseases represents a critical limitation for antibody detection technologies (12). In recent years, nucleic acid amplification diagnostics have emerged as a pivotal tool for pathogen detection, leveraging advantages such as rapidity, high sensitivity, and specificity (13). Notwithstanding, the most widely adopted technique, real-time PCR, can only detect a maximum of 4–6 pathogens per reaction tube. Given the vast diversity of respiratory pathogens (encompassing hundreds of species) and the frequent occurrence of mixed infections, the development of ultra-high-throughput detection methods for respiratory pathogens is urgently warranted.

High-throughput sequencing technologies, particularly metagenomic next-generation sequencing (mNGS) and targeted next-generation sequencing (tNGS), have emerged as powerful platforms for pathogen detection in clinical samples. mNGS holds the potential to identify all pathogens present in a clinical sample, yet the large amount of host genomic data often compromises its sensitivity for pathogen detection (14). By contrast, tNGS offers enhanced speed, sensitivity, and cost-effectiveness compared to mNGS, as it focuses on analyzing specific targets of clinical relevance (15, 16). tNGS employs either multiplex PCR amplicon or probe capture to enrich target pathogens. While amplicon sequencing boasts advantages like fewer workflow steps, operational flexibility, and reduced turnaround time, it faces notable challenges (17, 18). For instance, PCR amplification inherently introduces bias, leading to unequal target enrichment. Additionally, an increase in primer pairs heightens the risk of primer dimer/multimer formation, which can diminish amplification efficiency or even cause amplification failure. Furthermore, Taq enzyme-mediated errors during amplification necessitate careful discrimination between genuine genetic variations and sequencing artifacts. Lastly, delayed primer updates may undermine the efficacy of amplicon sequencing in detecting emerging mutations (19). In comparison, capture sequencing offers distinct benefits. These include higher target number per panel, superior sequence uniformity, higher fault tolerance, and reduced risk of aerosol contamination.

However, the traditional liquid-phase hybrid capture sequencing process is highly time-consuming and cumbersome, requiring 2–3 days from nucleic acid extraction to capture library construction (20). To address the limitations of conventional liquid-phase hybrid capture, our previous study developed a novel, rapid, and efficient Micro-Targets Hybrid Capture (MT-Capture) system, which has been successfully applied to SARS-CoV-2 whole-genome sequencing (21). Although respiratory multi-pathogen capture sequencing differs in purpose from whole-genome sequencing, their experimental workflows, especially hybridization process, share consistency—both employ hybrid capture for library molecules of similar sizes. Thus, in this study, we developed a novel assay (RP-MT-Capture NGS) for detecting multiple respiratory pathogens (RP) by integrating MT-Capture technology with NGS. By optimizing probe design and hybridization capture procedures, RP-MT-Capture NGS achieved high detection sensitivity and remarkably short operation time (within 6 h for wet lab experiment). Compared with the MT-Capture whole-genome sequencing assay for SARS-CoV-2, the RP-MT-Capture NGS assay established in this study has two fundamental innovations. First, it enables the simultaneous detection of over 300 species/types of respiratory pathogens, whereas the former can only detect the coronavirus genome. Second, this assay can additionally obtain the full-length sequences of hemagglutinin (HA) and neuraminidase (NA) gene segments of influenza viruses, providing a reliable tool for influenza virus mutation surveillance and recombination analysis. Clinical evaluation of RP-MT-Capture NGS has demonstrated that the assay delivers exceptional detection performance and holds substantial promise for the identification of multiple respiratory pathogens.

## RESULTS

### The workflow and panel design principles of RP-MT-Capture NGS

**Fig 1:**
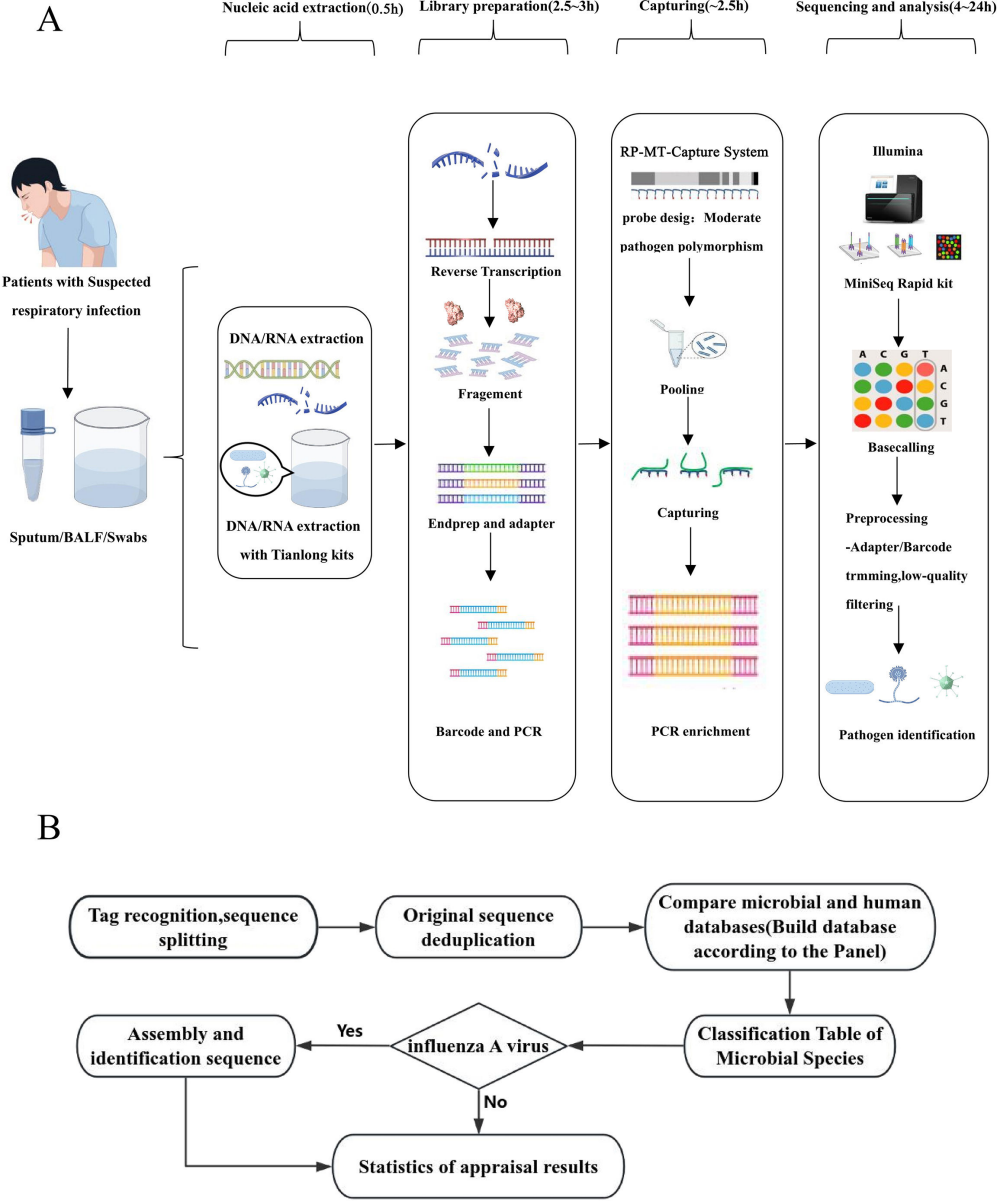
The workflow and bioinformatics analysis of RP-MT-Capture NGS. (**A**) The overall detection process of RP-MT-Capture NGS assay. (**B**) The bioinformatics analysis pipeline. BALF, bronchoalveolar lavage fluid.

A comprehensive overview of the RP-MT-Capture NGS workflow and bioinformatics analysis is illustrated in Fig. 1. MT-Capture can be completed within 2.5 h, resulting in the wet-lab operations of RP-MT-Capture NGS finishing within 6 h. Compared with mNGS, RP-MT-Capture NGS offers reduced costs and enhanced sensitivity. In contrast to the TaqMan array, this assay provides extensive coverage of major respiratory pathogens and enables high-throughput analysis of more samples in a single run. A detailed comparison of the performance characteristics of the three methods is presented in Table 1.

**TABLE 1:** Comparison of performance characteristics among three common multiplex pathogen detection technologies

Performance metric		mNGS	MT-Capture NGS	Taqman array
Cost		High	low	low
Covered pathogens		Extensive	Moderate	Limited
Sensitivity	For Bacteria	Moderate	High	High
	For virus	Low	High	High
Wet lab experiment		5 h	6 h	3 h
Technical bottlenecks		Co-infection interference (high genomic similarity microorganisms)；Host genomic interference and Competitive interference; The voluminous sequencing data presents a significant challenge in terms of analysis complexity.	The detection sensitivity is correlated with the size of the target region covered by the probe, and the probe must take into account the polymorphism of the target.	Chip throughput limits the number of detectable pathogens and samples; Pathogen genetic variation poses a risk of missed detection.

**Fig 2:**
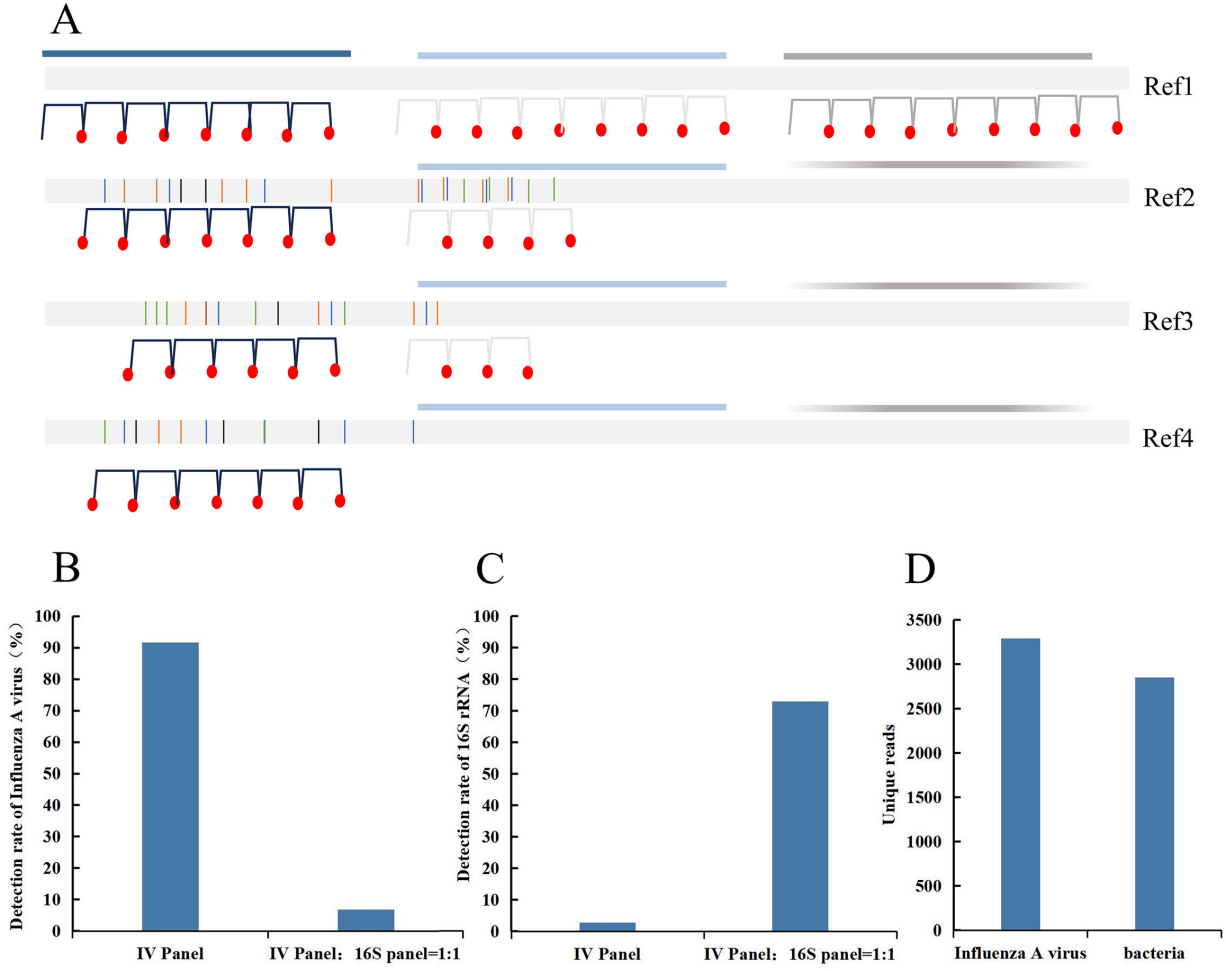
MT-Capture probe design strategy. (**A**) The relationship between the number of MT-Capture probes designed and the conservation of target regions. (**B and C**) The proportion of reads for influenza A (**B**) and 16S rRNA (**C**) when a sample co-infected with influenza A virus and bacteria was detected using MT-Capture probes targeting both the influenza virus and 16S rRNA (IV panel: 16s panel = 1:1) or only influenza virus (IV panel). (**D**) The sequencing results obtained by detecting a sample co-infected with influenza A virus and bacteria using MT-Capture probes, which target influenza virus and bacterial-specific genes other than 16S rRNA.

Given the diversity and complexity of respiratory pathogens, probe design should prioritize highly conserved genomic regions (Fig. 2A). This strategy not only cuts down probe costs but also boosts the efficiency of pathogen capture. RNA pathogens typically have fewer conserved regions. For these pathogens, it is recommended to select prevalent strains as reference genomes and moderately increase probe density to enhance capture efficiency. The 16S ribosomal RNA (rRNA), 18S rRNA, and the internal transcribed spacer (ITS) regions are frequently used as molecular markers for identifying bacterial or fungal species. However, the multicopy nature of these genes may render them less suitable as targets for RP-MT-Capture NGS. For example, designing probes for 16s region will capture more ribosomal targets, which can sequester the data of low-abundance pathogens and affect the detection rate of these pathogens. Our results confirmed that targeting 16s rRNA substantially compromised the detection sensitivity of RNA viruses (Fig. 2B and C). Therefore, we selected specific genes other than 16S, 18S, and ITS as targets for the design of bacterial or fungal probes (Fig. 2D; Table S1). Considering the detection sensitivity and cost-effectiveness, we used conserved regions of approximately 500 base pairs for probe design. Due to the epidemiological significance and unique characteristics of influenza A virus, its probe set was designed to target the full-length sequences of both the HA and NA genes.

### Analytical sensitivity of RP-MT-Capture NGS

**Fig 3:**
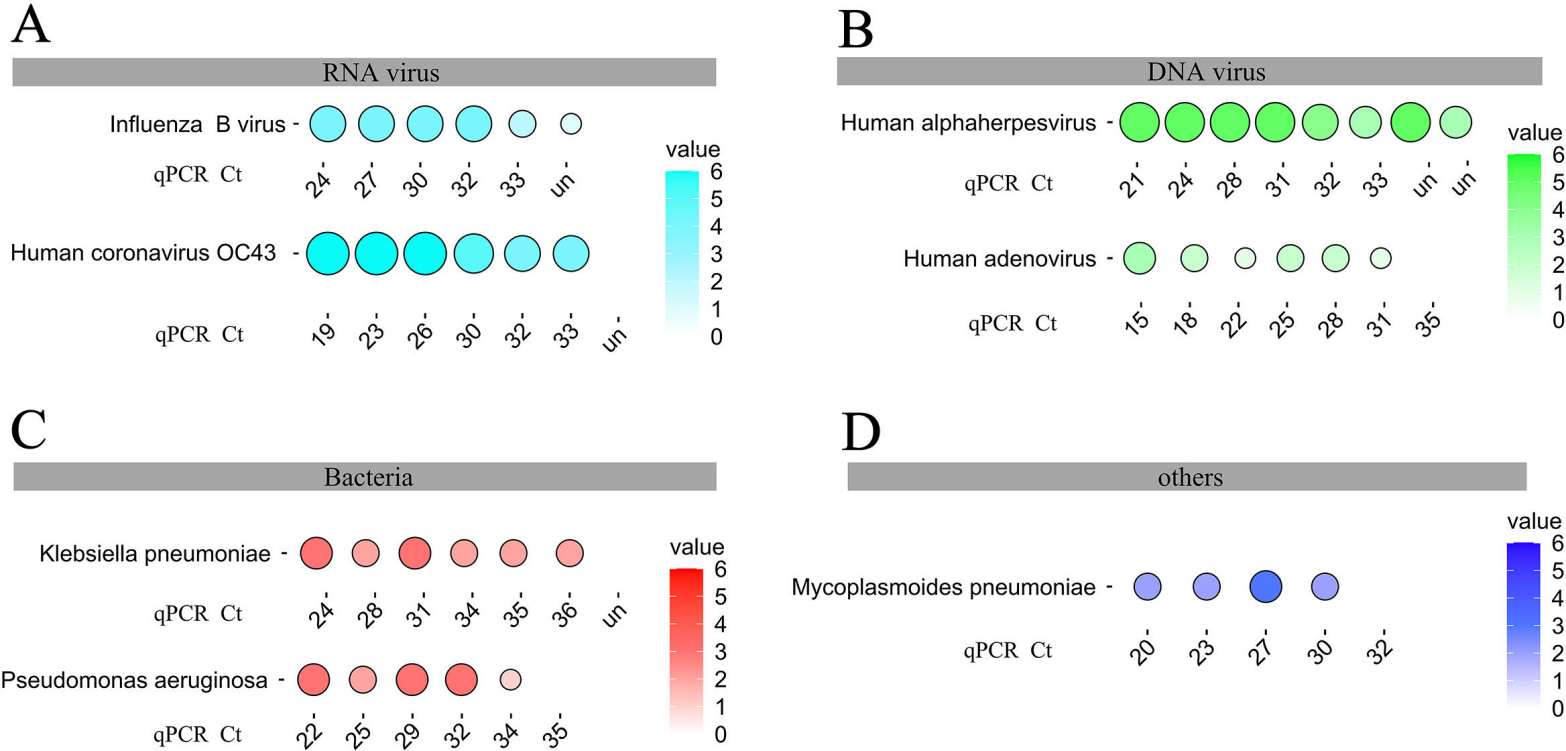
Analytical sensitivity of RP-MT-Capture NGS assessed by representative pathogens. (**A–D**) The results of RP-MT-Capture NGS detecting serially diluted nucleic acids extracted from representative respiratory samples positive for RNA viruses (**A**), DNA viruses (**B**), bacteria (**C**), or *Mycoplasma pneumoniae* (**D**). For influenza B virus and human alphaherpesvirus, RP-MT-Capture NGS can detect samples with CT values > 33; for human coronavirus OC43, Klebsiella pneumoniae, Pseudomonas aeruginosa, human adenovirus, and Mycoplasmoides pneumoniae, RP-MT-Capture NGS can detect samples with CT values of 33, 36, 34, 31, and 30, respectively. Both the size and color intensity of the circles represent the value of log_10_^(Unique RPM)^.

The analytical sensitivity of RP-MT-Capture NGS was evaluated by testing serially diluted nucleic acid extracted from respiratory samples positive for different kinds of pathogens. The assay successfully detected influenza B virus, human coronavirus OC43, and human alphaherpesvirus in samples with CT values ≥ 33, as well as Klebsiella pneumoniae and *Pseudomonas aeruginosa* at CT values of 36 and 34, respectively (Fig. 3). These results demonstrate that the RP-MT-Capture NGS assay exhibits high analytical sensitivity.

### High-precision pathogen subtyping and full-length detection of HA/NA genes of influenza virus

The RP-MT-Capture NGS assay developed in this study can not only identify the species but also characterize the types of many common pathogens. As shown in Table 2, the assay accurately identified all the types of tested viruses. Additionally, it enabled multiplex detection of pathogens in a single reaction. For example, sample ML8 showed co-infection with human respiratory syncytial virus A, human orthopneumovirus, and human coronavirus OC43 (Table 2).

**TABLE 2:** The pathogen typing results of RP-MT-Capture NGS

Pathogens	Sample ID	Diagnostic results (Real-time PCR)	RP-MT-Capture NGS results		
			Typing	Unique reads	Unique RPM
Influenza A virus	ML4	Influenza A virus H5N1	Influenza A virus H5N1 H	13617	18351.0
			Influenza A virus H5N1 N	6117	8243.0
	ML5	Influenza A virus H7N9	Influenza A virus H7N9 H	175898	24577.0
			Influenza A virus H7N9 N	163978	22912.0
Human Respiratory Syncytial Virus	ML8	Human Respiratory Syncytial Virus A	Human Respiratory Syncytial Virus A	14176	56601.2
			Human orthopneumovirus	1075	4292.2
			Respiratory syncytial virus	159	634.8
			Human coronavirus OC43	1	4.0
	TAC-139	Human Respiratory Syncytial Virus B	Human Respiratory Syncytial Virus B	18	31.8
			Stenotrophomonas maltophilia	91	161.0
			Pseudomonas aeruginosa	16	28.3
			Bordetella bronchiseptica	4.0	7.1
			Nocardia asteroides	1.0	1.8
Human Parainfluenza Virus	ML10	Human Parainfluenza Virus 2	Human Parainfluenza Virus 2	119342	65971.0
			Human gammaherpesvirus 4	1	1
			Human coronavirus HKU1	1	1
	ML11	Human Parainfluenza Virus 3	Human parainfluenza virus 3	21295	13085.9
			Human gammaherpesvirus 4	5	3.1
	ML12	Human Parainfluenza Virus 4	Human parainfluenza virus 4	10455	63048.0
			Human coronavirus OC43	2	12.1
Human Adenovirus	ML25	Human Adenovirus 7	Human Mastadenovirus B	47677	70493.8
			Human adenovirus 16	182	269.1
			Human Adenovirus 7	500	739.3
	TAC-154	Human Adenovirus 5	Human Mastadenovirus C	14	9.0
			Human Adenovirus 5	26	46.0
			Human Gammaherpesvirus 4	78	137.8
			Stenotrophomonas maltophilia	147	259.8
			Pseudomonas aeruginosa	13	23.0
			Klebsiella pneumoniae	5	8.8
			Human metapneumovirus	3	5.3
			Bordetella bronchiseptica	3	5.3
			Neisseria meningitidis	1	1.8

**Fig 4:**
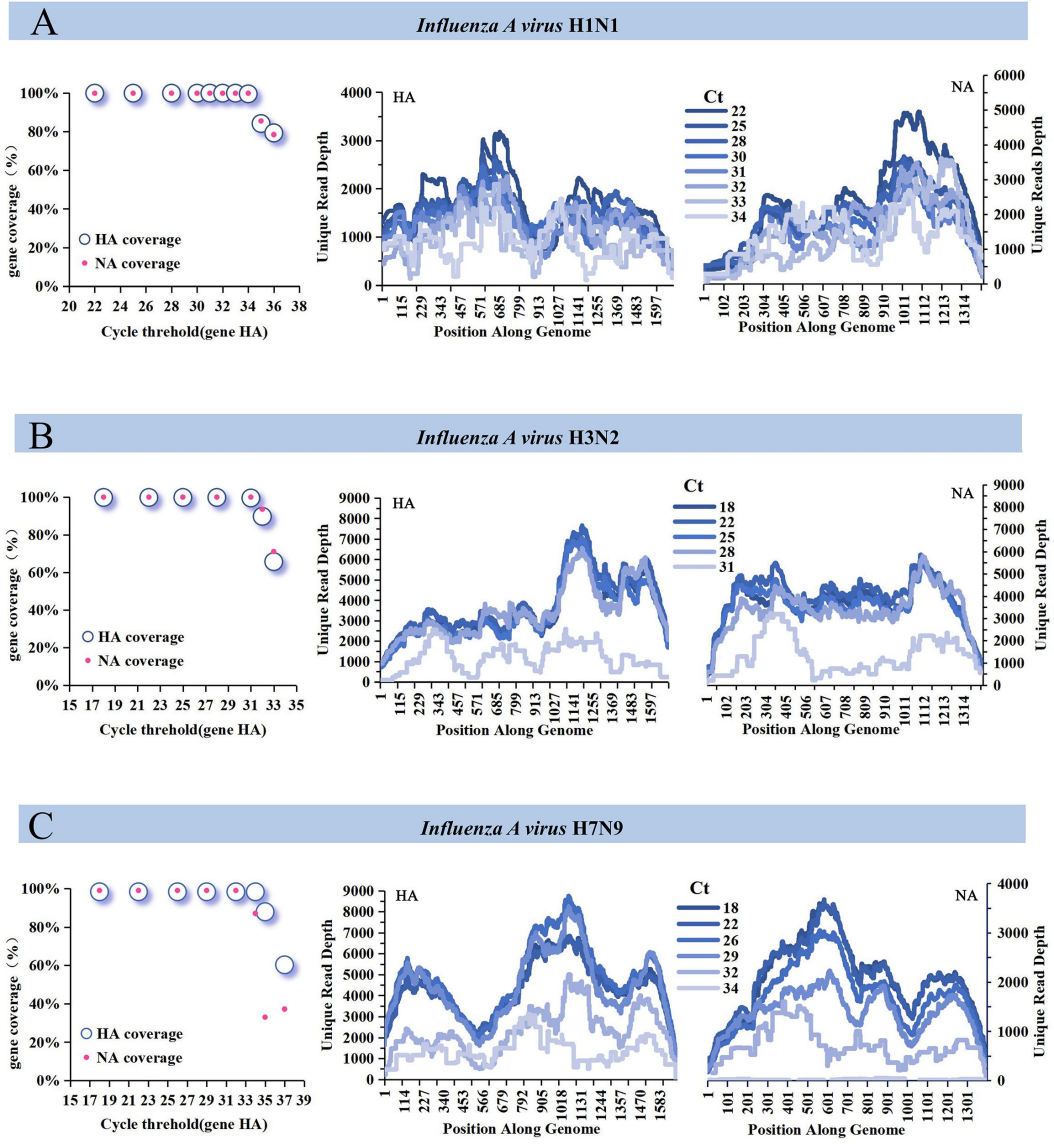
The detection performance for the full length of HA/NA gene segments of influenza A virus by RP-MT-Capture NGS. (**A–C**) The gene coverage and Unique Read Depth of serially diluted samples positive for H1N1 (**A**), H3N2 (**B**), and H7N9 (**C**) viruses with different CT values.

Furthermore, RP-MT-Capture NGS enables full-length detection of HA/NA genes of influenza virus, facilitating the monitoring of viral mutations and recombination events. Serial dilutions of H1N1, H3N2, and H7N9 positive samples were analyzed in parallel using RP-MT-Capture NGS and real-time PCR. Results confirmed that RP-MT-Capture NGS accurately identified all tested influenza subtypes. Notably, for samples with CT values < 32, the HA and NA gene coverage reached 100% (Fig. 4). The phylogenetic analysis revealed that the H1N1 HA gene belongs to clade 6B.1A and the H3N2 HA gene belongs to clade 3C.2a1b.2 (Fig. S1). The high sensitivity of HA/NA sequencing is valuable for both epidemic prevention and control and clinical treatment guidance.

### RP-MT-Capture NGS for clinical sample analysis

**Fig 5:**
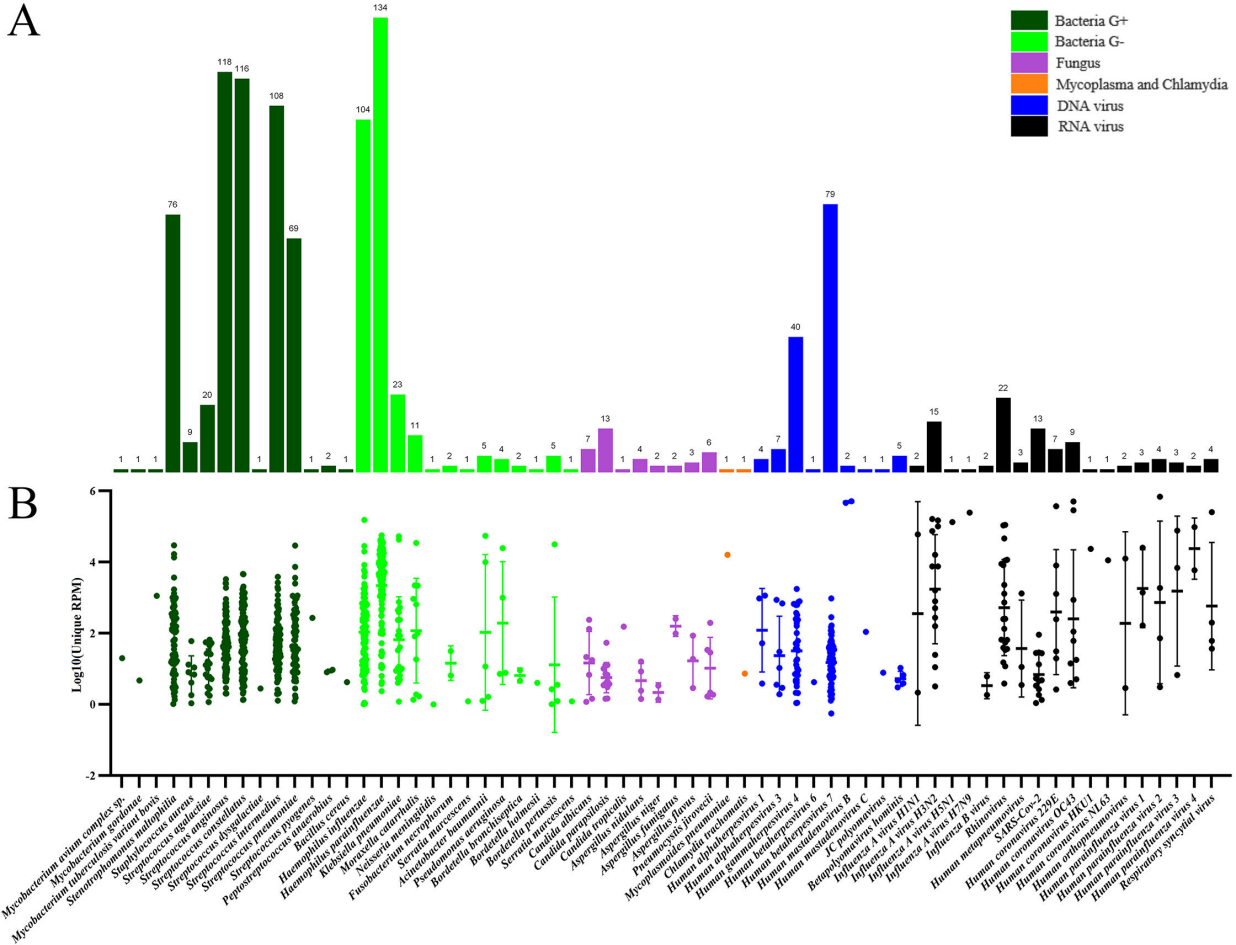
The pathogen identification results of 186 clinical samples obtained by RP-MT-Capture NGS. (**A**) The number of positive samples for each detected target. (**B**) The relative abundance of positive targets in each sample, expressed as log_10_^(Unique RPM)^.

A total of 186 respiratory samples from acute respiratory infection (ARI) patients were analyzed using the RP-MT-Capture NGS assay. As shown in Fig. 5, 64 distinct pathogens were detected, comprising 14 kinds of Gram-positive bacteria, 13 kinds of Gram-negative bacteria, 8 kinds of fungi, 9 kinds of DNA viruses, 18 kinds of RNA viruses, 1 kind of mycoplasma, and 1 kind of chlamydia each. The most prevalent bacteria were Haemophilus parainfluenzae, Streptococcus anginosus, Streptococcus constellatus, Streptococcus intermedius, Haemophilus influenzae, Stenotrophomonas maltophilia, and Streptococcus pneumoniae. The top five viruses included Human betaherpesvirus 7 (Roseolovirus), Human gammaherpesvirus 4 (EB virus), Rhinovirus, Influenza A virus H3N2, and SARS-CoV-2.

**Fig 6:**
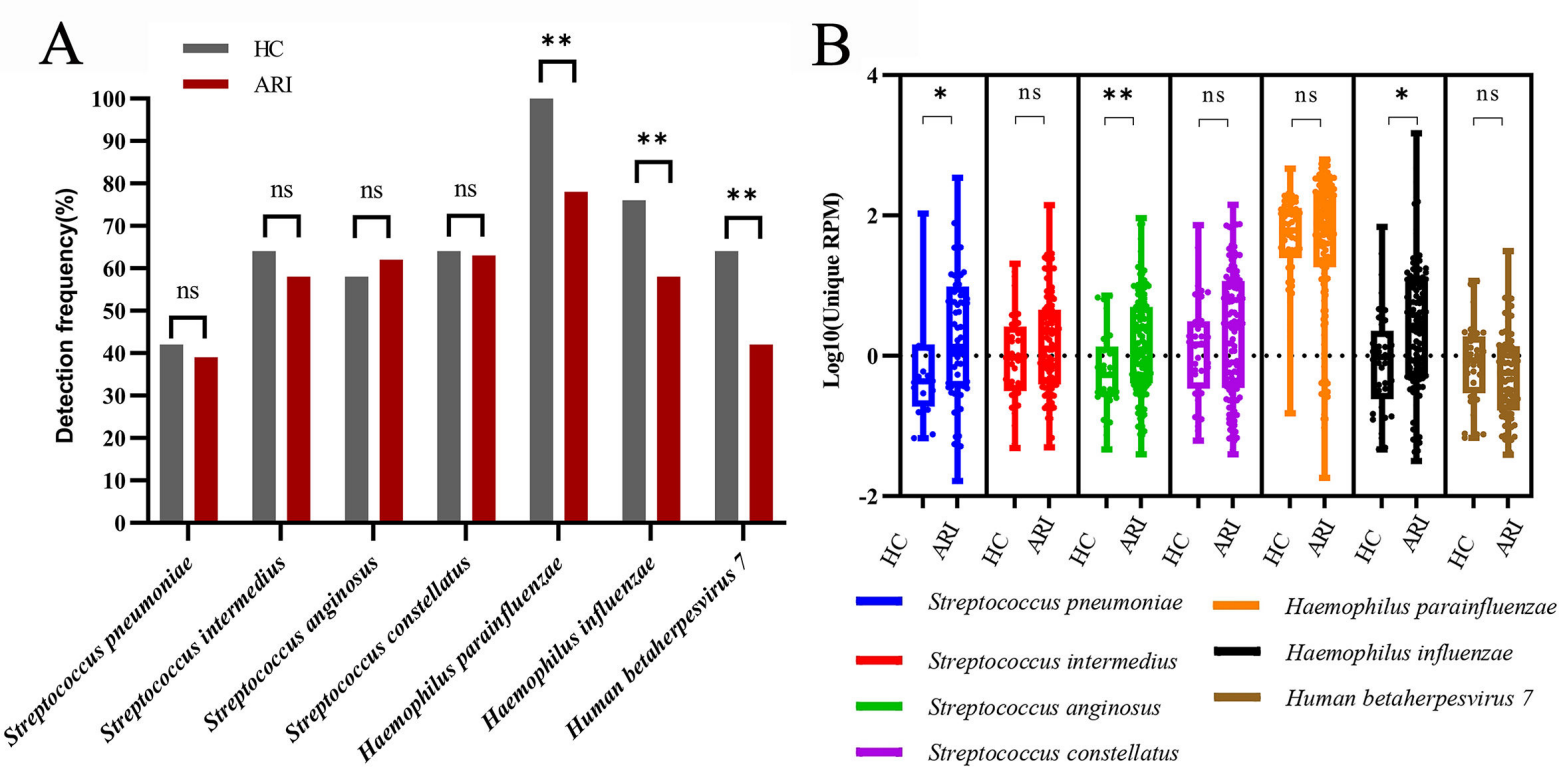
The carriage characteristics of the seven opportunistic pathogens in HC and ARI samples. (**A**) The detection rates of seven opportunistic pathogens in HC and ARI samples. Differences between groups were compared using the chi-square test. (**B**) The relative abundances of seven opportunistic pathogens in each HC or ARI sample, expressed as log_10_^(Unique RPM)^. The nonparametric Mann-Whitney *U* test was used to compare the differences between groups. * and ** stand for *P* < 0.05 and *P* < 0.01, respectively. ns stands for not significant.

Many detected pathogens (particularly bacteria) mentioned above are opportunistic pathogens. To assess the carriage characteristics of these pathogens in healthy populations, RP-MT-Capture NGS was conducted on samples obtained from 50 healthy controls (HCs). Results showed that the detection rates of seven opportunistic pathogens were either approaching or exceeding 50% (Fig. 6A). We further compared the detection rates and relative abundances of the seven pathogens in ARI and HC samples. Results showed that the detection rates of these pathogens in ARI samples were not significantly different from, and in some cases even lower than, those in HC samples, suggesting that in some ARI patients, infection with other specific pathogens might suppress the colonization of these opportunistic pathogens (Fig. 6A). The results of pathogen loads confirmed this hypothesis; as shown in Fig. 6B, the relative abundances of these pathogens in some ARI samples were significantly lower than those in the HC samples. However, overall, the distribution ranges of the relative abundances of these pathogens in ARI samples were broader, and the average pathogen loads in positive ARI samples were higher (Fig. 6B). Streptococcus pneumoniae, Streptococcus anginosus, and Haemophilus influenzae showed significantly higher pathogen loads in ARI samples, suggesting these pathogens might proliferate secondarily due to decreased immune function, leading to disease. Taken together, these findings suggest that when interpreting positive results for opportunistic pathogens, it is essential to integrate clinical manifestations with the relative abundances of pathogens to determine their potential pathogenicity.

### RP-MT-Capture NGS exhibits superior accuracy and sensitivity compared to TaqMan array and mNGS for respiratory pathogen detection

**Fig 7:**
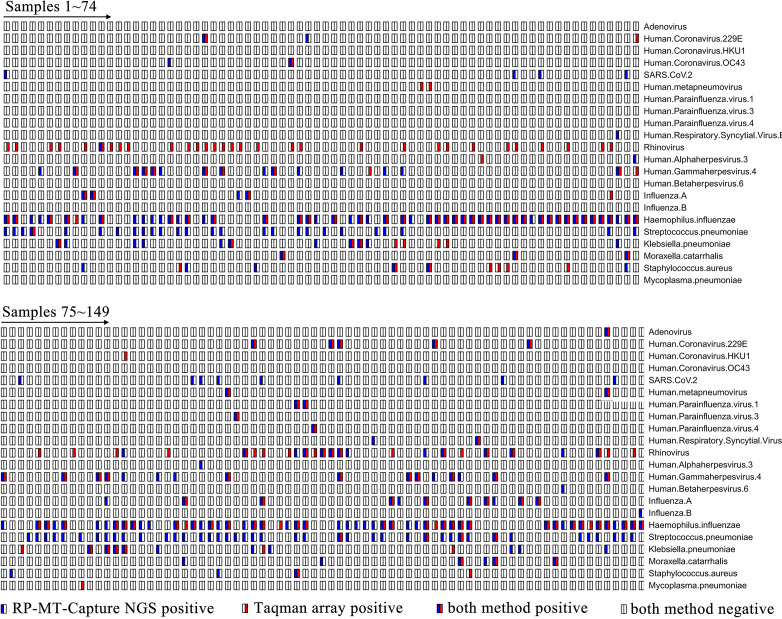
Detection results of RP-MT-Capture NGS and Taqman array for 149 ARI samples positive for at least one pathogen. Red color indicates pathogens identified by the TaqMan array, and blue color indicates pathogens identified by RP-MT-Capture NGS.

**Fig 8:**
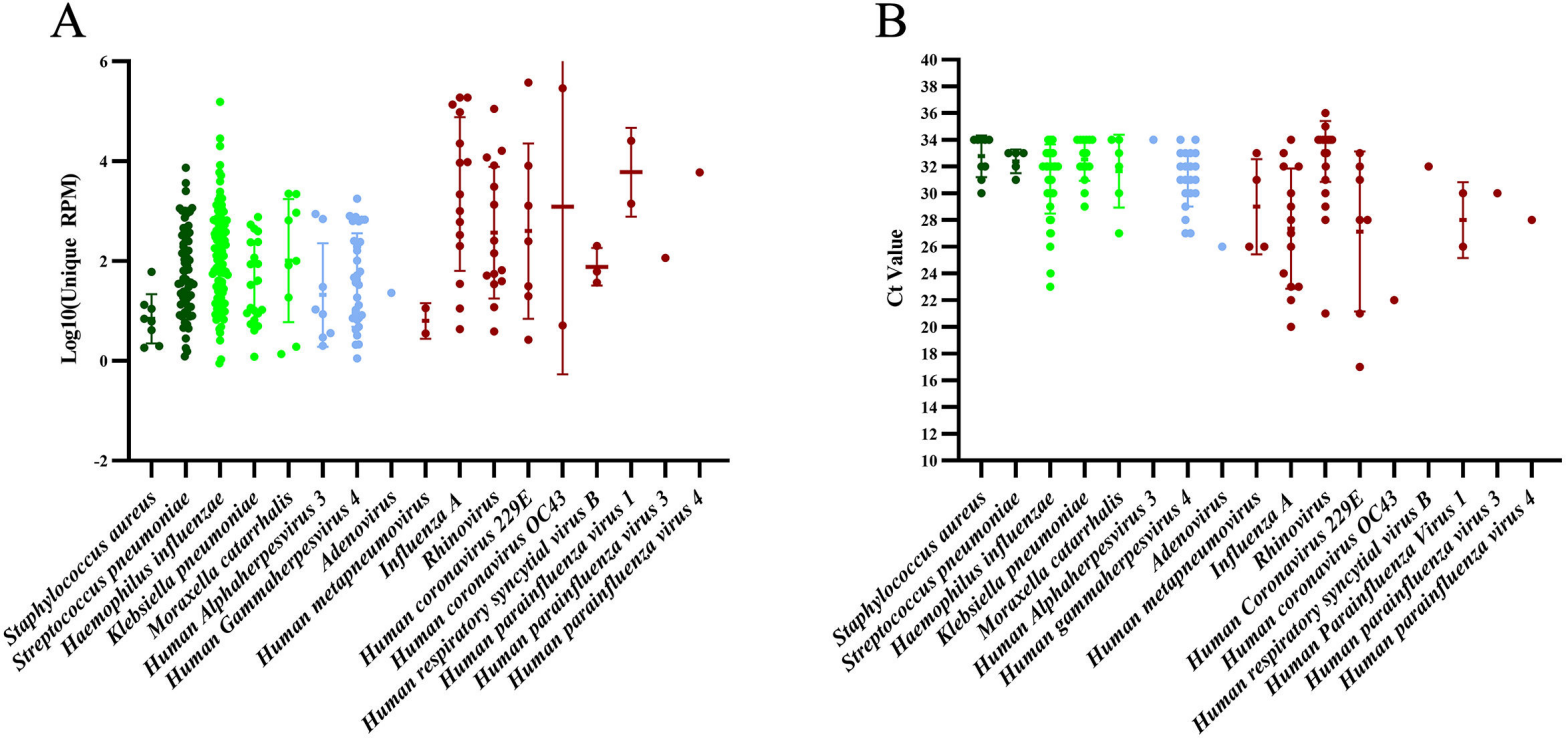
Detection results of co-detected pathogens by both RP-MT-Capture NGS and TaqMan array. (**A**) RP-MT-Capture NGS detection results for the 17 co-detected pathogens, where the scatter points represent the relative abundances of pathogens in the positive samples. (**B**) TaqMan array detection results for the 17 co-detected pathogens, where the scatter points represent the CT values of pathogens in the positive samples.

One hundred and fifty-nine ARI samples were randomly selected from 186 samples that had undergone RP-MT-Capture NGS testing for parallel analysis using the TaqMan array. A comparative evaluation was conducted on the detection results of 41 common respiratory pathogens covered by both methods. Out of the 159 samples, no pathogen (among the 41 pathogens) was detected by either method in 10 samples, while at least 1 pathogen was identified in the remaining 149 samples (Fig. 7). The detection rate for RP-MT-Capture NGS was 95.97% (140/149), compared to 84.56% (126/149) for the TaqMan array. Among the 149 samples, we identified 22 pathogens, 17 of which were co-detected by both techniques. Except for rhinovirus, RP-MT-Capture NGS identified a significantly higher number of positive samples for most of the co-detected pathogens than the TaqMan array (Fig. 8).

In addition to the co-detected pathogens, among the 41 pathogens, 3 pathogens (SARS-CoV-2, Human betaherpesvirus 6, and Influenza B virus) were exclusively detected by RP-MT-Capture NGS (involving 15 samples), and 2 pathogens (Human coronavirus HKU1 and Mycoplasma pneumoniae) were exclusively detected by the TaqMan array (involving 2 samples, with both CT values ≥ 33). Both Real-time PCR and BLAST sequence alignment analyses of the detected sequences confirmed all the positive outcomes (Tables S2 and S3; Fig. S2). These data indicate that, for most respiratory pathogens, the RP-MT-Capture NGS offers superior performance compared to the TaqMan array. However, for samples of Human coronavirus HKU1 and Mycoplasma pneumoniae with low viral load, the TaqMan array shows better detection performance.

To further compare the performance of RP-MT-Capture NGS and mNGS in detecting respiratory pathogens, 38 specimens with relatively high pathogen loads as detected by RP-MT-Capture NGS were selected from the 186 ARI samples and subjected to mNGS. Results showed that mNGS achieved a detection rate of 73.1% for viruses, 100% for bacteria, and 0% for fungi, as compared to those of RP-MT-Capture NGS (Table 3). Further analysis showed that viral enrichment folds of RP-MT-Capture NGS ranged from 463 to 11,801 (Table S4).

**TABLE 3:** The positive and negative agreement rates between RP-MT-Capture NGS and mNGS in detecting respiratory pathogens among 38 samples

Positive by RP-MT-Capture NGS (38 samples)	mNGS		
	Positive	Negative	Agreement (%)
Viruses^*a*^ (26 samples)	19	7	73.1
Bacteria^*a*^ (11 samples)	11	0	100
Fungi^*a*^ (1 sample)	0	1	0

^*a*^ The results were only analyzed based on the respiratory pathogens covered by the RP panel.

## DISCUSSION

Respiratory infections are common diseases caused by diverse pathogens, including bacteria, viruses, fungi, mycoplasmas, and chlamydia. These pathogens exhibit high diversity in types and complex transmission patterns; notably, distinct pathogens can induce similar clinical manifestations, thereby increasing diagnostic and therapeutic challenges (22, 23). Furthermore, respiratory pathogens frequently involve co-infections, and certain RNA viruses display high mutation rates, which further complicate diagnosis (24). Thus, rapid and accurate pathogen identification is critical for improving prognosis, reducing mortality, and controlling infectious disease spread. Nucleic acid amplification tests represented by real-time PCR have become important tools for pathogen detection due to their rapidity, sensitivity, and specificity. Various forms of multiplex real-time PCR and TaqMan array enable simultaneous detection of multiple pathogens. However, these methods either have a limited number of detection targets or a low sample throughput per test.

mNGS is an effective high-throughput molecular detection technology for multiple pathogens; however, this assay has limitations such as host interference, low sensitivity, high cost, and long turnaround time (25, 26). Amplicon-based tNGS, despite high sensitivity, fails to cope with pathogen variations and cross-interference with excessive targets (27, 28). In this study, we developed a novel RP-MT-Capture NGS assay, which enables simultaneous identification of over 300 respiratory pathogen species/types with high throughput and sensitivity. The high error tolerance of probes enables this assay to easily cope with pathogen variations. Compared with that of the time-consuming traditional probe hybridization-based tNGS (20), RP-MT-Capture NGS significantly shortens wet-lab experiment time (within 6 h).

While 16S rRNA is an ideal target for bacterial species identification (29), multicopy genes may not be suitable as detection targets for RP-MT-Capture NGS based on its detection principle. This is because when bacteria and RNA viruses coexist in a sample, 16S rRNA is likely to dominate most sequencing data, thereby impairing viral detection performance. Our experimental results have confirmed this hypothesis. Therefore, for all bacterial and fungal pathogens, we excluded multicopy genes such as 16S, 18S, and ITS as detection targets, and instead designed probes based on other pathogen-specific genes. This strategy helps enhance RNA virus detection while ensuring accurate identification of bacteria and fungi.

Among respiratory pathogens, influenza virus is highly variable, and its classification depends on two key proteins: HA and NA (30). Influenza viruses undergo major genetic variations such as antigenic shift every decade or so, which may give rise to new strains. These strains pose a significant potential threat to humans, making it necessary to continuously track and monitor the variations of influenza virus. Most reported respiratory multi-pathogen detection technologies based on NGS platforms can only identify pathogens but fail to monitor the variation of influenza viruses (31, 32). The RP-MT-Capture NGS assay established in this study can detect full-length HA and NA genes, offering a powerful tool for monitoring viral mutations and recombination analysis. Furthermore, amplicon-based tNGS has limited scalability. Due to cross-interference between multiplex PCR primer pairs, if the existing detection panel needs to be updated, for example, to include additional pathogens, the newly added primer pairs may affect the efficiency of previously optimized primers. Consequently, the entire detection system may require re-optimization and re-evaluation. In contrast, the RP-MT-Capture NGS assay established in this study offers significant advantages in updating detection panels. Simply adding new pathogen-specific probes to the original probe panel allows for easy updates, enabling the detection of newly emerging pathogens. In terms of cost-effectiveness, detecting >300 respiratory pathogens per sample costs ~70 US dollars, significantly lower than mNGS costs.

For detecting opportunistic pathogens, the lack of well-defined virulence genes complicates distinguishing their infection from colonization (33–35). Following primary infection in ARI patients, opportunistic pathogens may exhibit two scenarios: first, the proliferation of primary pathogens inhibits the growth of opportunistic pathogens in some individuals, which may lead to a decreased detection rate of opportunistic pathogens (Fig. 6A); second, primary infection-induced disruption of the body’s barriers and immune balance results in secondary proliferation of opportunistic pathogens in some individuals, which may cause an increased pathogen loads in positive individuals (Fig. 6B). Our results suggest that when interpreting positive results for opportunistic pathogens, it is essential to integrate clinical manifestations with the abundances of the pathogens. The higher the abundance, the greater the potential association with the current disease. Standardized thresholds for the abundance of opportunistic pathogens in healthy controls will aid clinicians in result interpretation but require future multicenter large-scale studies and time to accumulate relevant data.

The RP-MT-Capture NGS assay was primarily developed to address the limitations of existing high-throughput detection technologies (e.g., high cost and low sensitivity of mNGS, as well as limited numbers of detection targets and samples for TaqMan array). Therefore, for performance evaluation, we mainly compared the detection performance of RP-MT-Capture NGS with that of mNGS and TaqMan array, rather than with traditional detection technologies. Although RP-MT-Capture NGS outperforms mNGS in detection sensitivity and TaqMan array in sample detection throughput, it still has certain limitations. First, the pathogen detection capability relies on the designed RP panel and cannot achieve full coverage of all pathogens. For instance, regarding rhinoviruses with over 120 serotypes, the RP panel cannot identify all the subtypes of rhinoviruses (36). This is related to the size of the target regions covered by the probes and the annotation of the reference genomes in the database. Some reference genomes are not clearly typed. In the future, the coverage of multi-type pathogens (such as rhinoviruses) can be improved by increasing probe density or optimizing database annotation. Second, the evaluation of the performance of RP-MT-Capture NGS in clinical sample detection was based on a relatively small sample size, which was also from a single research center. This may compromise the representativeness and generalizability of the study findings. Currently, we have begun to conduct multi-center validation of this assay to establish robustness. Finally, the library preparation and MT-Capture processes in this study were performed manually, involving numerous experimental steps. Automation of the workflow through an automated library preparation instrument would further reduce experimental time, minimize human-induced errors, and improve the result stability.

Collectively, the RP-MT-Capture NGS assay developed in this study offers a novel technical tool for detecting multiple respiratory pathogens. It allows simultaneous detection of most common respiratory pathogens, with substantial clinical and public health relevance.

## MATERIALS AND METHODS

### Clinical samples

A total of 236 nasopharyngeal swab or sputum samples collected by Jiangsu Provincial Center for Disease Control and Prevention were included in this study, comprising 186 samples from acute respiratory infection (ARI) patients and 50 samples from healthy control (HC) subjects. All samples were stored at −80°C until use. All procedures conducted in this study involving human materials were approved by the Ethics Committee of Jiangsu Provincial Center for Disease Control and Prevention, and informed consent was obtained from each participant involved. All the experiments were carried out in accordance with the Declaration of Helsinki.

### Design of MT-Capture probes targeting respiratory pathogens

Multiple sequence alignment was carried out on species- or type-specific genes of respiratory pathogens. Subsequently, relatively conserved regions were selected to design the MT-Capture probes, collectively referred to as the respiratory pathogen panel (RP panel). These probes are non-isometric, with lengths ranging from 20 to 100 nucleotides. Our previously published research has described the innovative probe structure of MT-Capture, which consists of one target-binding region and two conjugated regions. This structure facilitates the formation of a “hand-in-hand” conjugation effect [22]. The respiratory pathogens covered by the RP panel and the targeted genes utilized for MT-Capture are detailed in Table S1. Moreover, during the methodology establishment phase, MT-Capture probes targeting the 16S rRNA of bacteria were also designed to compare detection efficiencies.

### Nucleic acid extraction

Nucleic acid extraction and purification were conducted in a Biosafety Level 2 (BSL-2) laboratory. Nucleic acid was extracted from 200 µL of each sample with a viral RNA/DNA extraction kit or a bacterial genomic DNA extraction kit (Xi’an Tianlong Biotechnology Co., Ltd, China) on the automatic nucleic acid extraction instrument GeneRotex 96 (Tianlong Biotechnology). The extracted nucleic acids were stored at −80°C for subsequent analysis.

### Library preparation, MT-Capture, and sequencing

RNA and DNA libraries were simultaneously prepared using the NadPrep RNA & DNA Co-Preparation Module (Nanjing Nanodigmbio Biotechnology Co., Ltd., China). Then MT-Capture was performed using the probes designed above and the μCaler Hybrid Capture Reagents (Nanodigmbio Biotechnology). The detailed procedures for library preparation, MT-Capture, and PCR amplification are described in the Supplementary Materials and Methods. The purified PCR products were finally sequenced using the Illumina NovaSeq High Output Reagent Cartridge (300 cycles) (Illumina) on the Illumina NovaSeq 6000 sequencing platform.

### Analytical sensitivity and pathogen typing capability

To assess the analytical sensitivity of the RP-MT-Capture NGS assay, representative positive respiratory clinical samples were selected, including RNA viruses (influenza B virus, human coronavirus OC43), DNA viruses (human Alphaherpesvirus, human adenovirus), bacteria (Klebsiella pneumoniae, Pseudomonas aeruginosa), and *Mycoplasma pneumoniae*. Nucleic acids were extracted from these samples, serially diluted, and analyzed using the RP-MT-Capture NGS assay, with parallel real-time PCR analysis for comparison. To evaluate the pathogen typing capability of the RP-MT-Capture NGS assay, clinical samples confirmed by real-time PCR to be positive for different subtypes of influenza viruses (H5N1 and H7N9), respiratory syncytial viruses (types A and B), human parainfluenza viruses (types 2, 3, and 4), and human adenoviruses (types 5 and 7) were selected for RP-MT-Capture NGS testing.

### Performance of full-length sequencing of influenza HA and NA genes

To evaluate the performance of the RP-MT-Capture NGS assay for full-length detection of influenza virus HA and NA genes, nucleic acids from clinical samples confirmed by real-time PCR to be positive for H1N1, H3N2, and H7N9 were selected. After serial dilution, the samples were analyzed using both RP-MT-Capture NGS and real-time PCR.

### Evaluation of RP-MT-Capture NGS with clinical specimens

To comprehensively assess the performance of RP-MT-Capture NGS in the identification of pathogens in clinical samples, we analyzed 236 samples, encompassing 186 acute respiratory infection (ARI) specimens and 50 healthy controls (HC). To further evaluate the detection accuracy of RP-MT-Capture NGS, 159 samples were randomly selected from the 186 ARI samples and subjected to parallel analysis by a TaqMan Respiratory Tract Microbiota Comprehensive Card (Thermo Fisher Scientific Inc., USA). The full panel of detectable pathogens for this card is detailed in Table S5. Discrepant results were resolved via real-time PCR reconfirmation. Additionally, among RP-MT-Capture NGS-positive samples, we chose 38 specimens with high pathogen loads for mNGS analysis. This enabled a direct comparison of pathogen identification efficacy between the two methods.

### Bioinformatics analysis

The sequencing data results were processed and analyzed via an automated analysis platform. The main workflow encompassed the following steps: tag identification and data splitting, matching data to corresponding samples; the data were aligned to the human reference (hg19) and classification reference database using Burrow-Wheeler Aligner (version 0.7.17-r1188), and human reads were filtered. For the establishment of the Reference Gene Database: first, based on the target genes of the designed probes, a BLAST search was conducted on NCBI, relevant reference genomes were downloaded, screened to remove sequences that did not meet the requirements, and then a reference gene database was constructed (Table S6). The sequencing data were compared with the constructed database to identify the matching reference genomes. Then, the sequence with the highest similarity was analyzed in combination with the pathogen list, so as to determine the type of detected pathogens.

In the sequencing data result report, Unique reads were defined as sequencing reads that uniquely and unambiguously align to a specific location in the reference sequences during the alignment process. Using this metric as the number of reads detected for a species provided a more reliable reflection of the true expression level or coverage of a specific gene or region, thereby avoiding noise introduced by multiple alignments. Subsequently, the number of detected Unique reads was normalized using RPM (Reads Per Million mapped reads), calculated as unique RPM = (unique reads × 10^6^)/total mapped reads. This normalization step further eliminated the impact of varying sequencing depths across samples, facilitating more accurate comparisons between samples. Finally, the value of log10 ^(Unique RPM)^ was calculated by taking the base-10 logarithm of the Unique RPM. This transformation converted the skewed data distribution into one that is closer to a normal distribution, reducing the influence of extreme values on the data and providing a better representation of the data distribution.

### Statistical analysis

All statistical analyses were performed with SPSS software version 23.0. Categorical variables were expressed as percentages and compared using the chi-square test. The nonparametric Mann-Whitney *U* test was used to compare the levels of the relative abundances of opportunistic pathogens between HC and ARI groups. *P* < 0.05 was considered statistically significant.

## Data Availability

The raw sequence data from clinical samples have been deposited in National Microbiology Data Center (NMDC) with accession number NMDC10020112 (https://nmdc.cn/resource/genomics/project/detail/NMDC10020112).

## SUPPLEMENTAL MATERIAL

Supplemental Figures spectrum.02908-25-s0001.docx Figures S1 and S2.

Supplemental Tables spectrum.02908-25-s0002.docx Tables S1-S5

Table S6 spectrum.02908-25-s0003.xlsx The reference genome database constructed in this study.

ASM does not own the copyrights to Supplemental Material that may be linked to, or accessed through, an article. The authors have granted ASM a non-exclusive, world-wide license to publish the Supplemental Material files. Please contact the corresponding author directly for reuse.
