# Supplementary material for: A novel targeted hybrid capture-NGS assay for sensitive detection of multiplex respiratory pathogens
Source: Microbiol Spectr. 2025 Nov 17;14(1):e02908-25. doi: 10.1128/spectrum.02908-25 (PMC12772336; doi:10.1128/spectrum.02908-25)
Supplement: Supplemental Figures — Figures S1 and S2. [file spectrum.02908-25-s0001.docx]

**
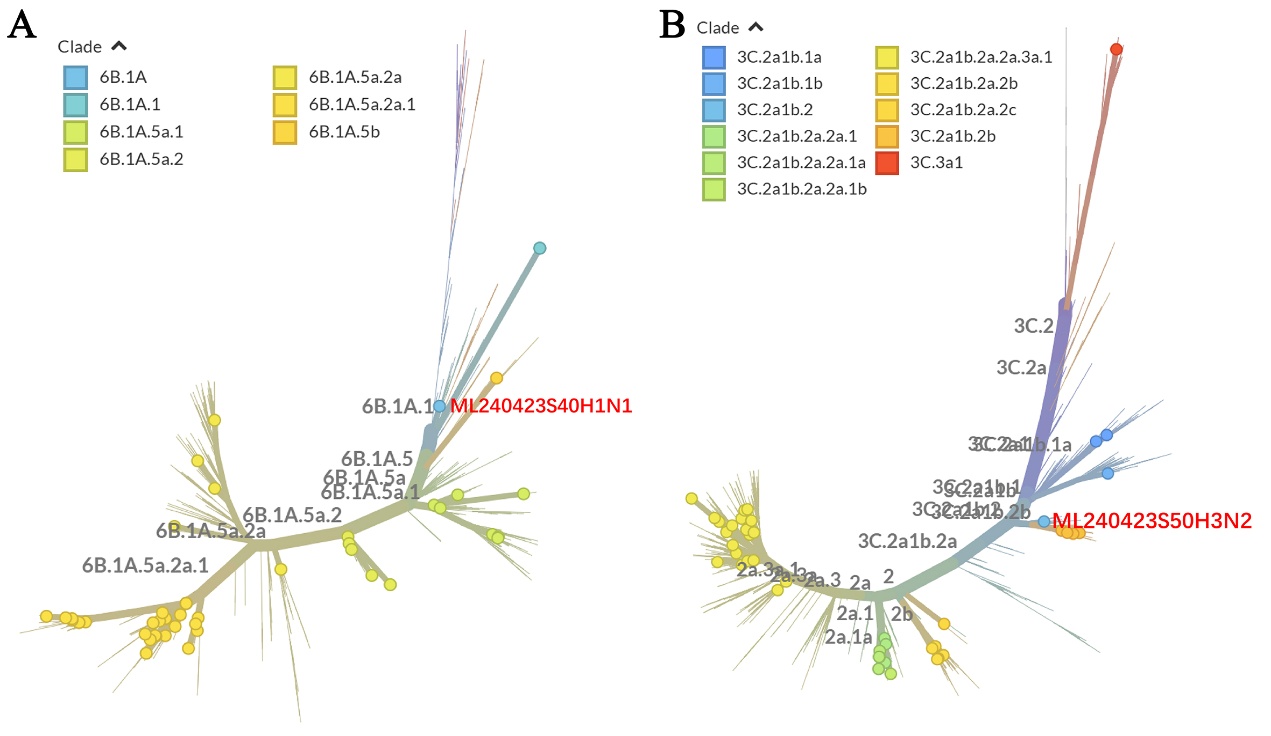
**

**SUPPLEMENTARY FIGURE 1** The phylogenetic analysis of HA gene segments of H1N1 and H3N2 influenza viruses. (A) The H1N1 HA gene belongs to clade 6B.1A (ML240423S40H1N1). (B) The H3N2 HA gene belongs to clade 3C.2a1b.2 (ML240423S50H3N2).

**
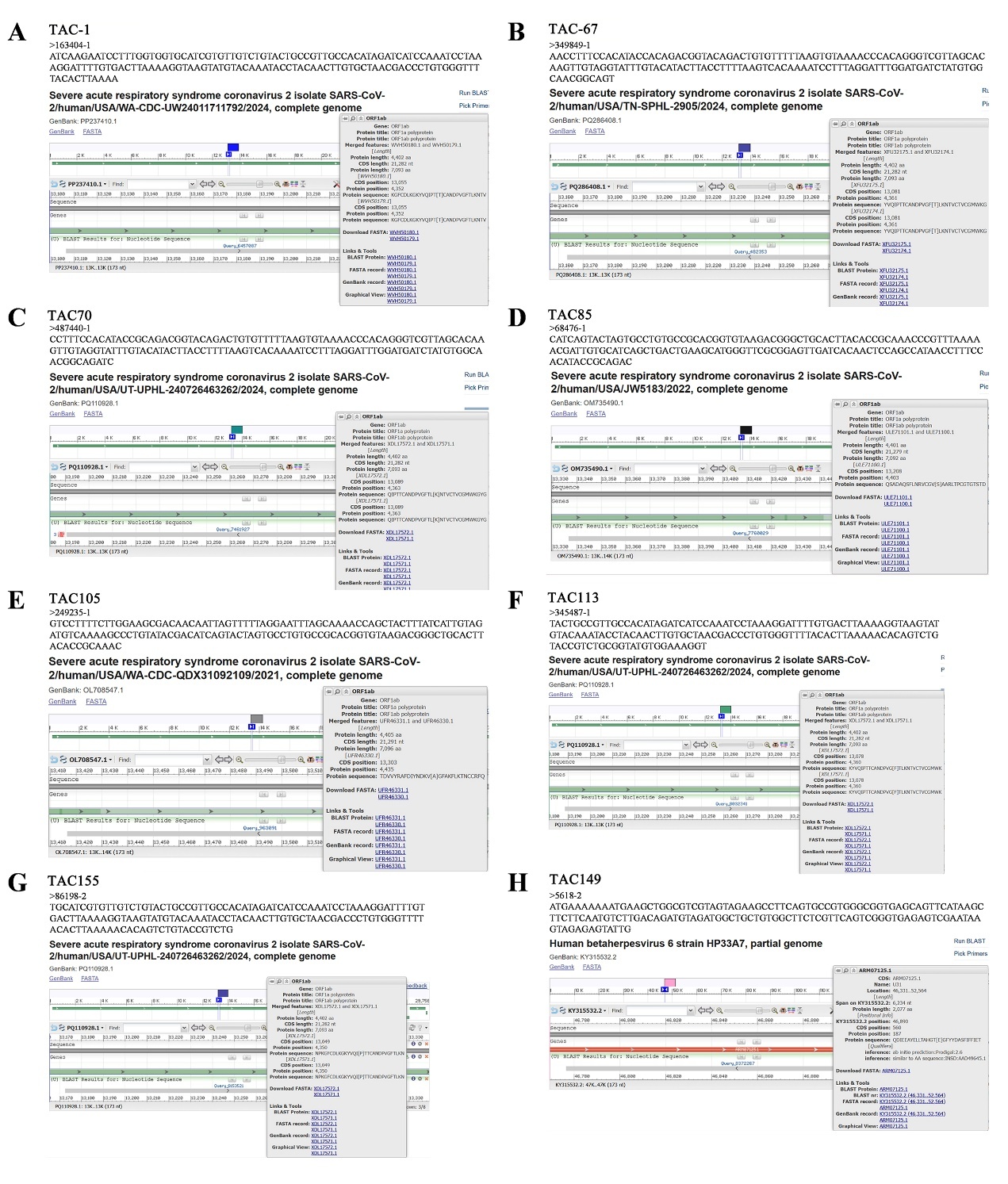
SUPPLEMENTARY FIGURE 2** BLAST sequence alignment analyses of samples that were positive for SARS-CoV-2 or human betaherpesvirus 6, exclusively detected by RP-MT-Capture NGS, but tested negative in Real-time PCR revalidation. Online BLAST sequence alignment analyses were performed on the reads identified as pathogen-positive exclusively by RP-MT-Capture NGS. (A-G) The alignment analysis results of SARS-CoV-2-positive samples (see Supplementary Table 3). H The alignment analysis results of human betaherpesvirus 6-positive sample (see Supplementary Table 3).
