# Supplementary material for: A novel targeted hybrid capture-NGS assay for sensitive detection of multiplex respiratory pathogens
Source: Microbiol Spectr. 2025 Nov 17;14(1):e02908-25. doi: 10.1128/spectrum.02908-25 (PMC12772336; doi:10.1128/spectrum.02908-25)
Supplement: Supplemental Tables — Tables S1-S5 [file spectrum.02908-25-s0002.docx]

**SUPPLEMENTARY TABLE 1** Pathogens covered by the RP panel and the targeted genes utilized for MT-Capture

| **Pathogen Name** | **Target gene** |
| --- | --- |
| *BK Polyomavirus* | *BKPyVgp3* |
| *JC Polyomavirus* | *Late mRNA gene* |
| *WU Polyomavirus* | *VP1* |
| *Human Mastadenovirus B (Human Adenovirus 3, 7, 11, 14, 16, 21, 34, 35, 50, 55)* | *Hexon* |
| *Human Mastadenovirus C (Human Adenovirus 1, 2, 5, 6, 57)* | *Hexon* |
| *Human Mastadenovirus D (Human Adenovirus 8, 27, 28, 30，38, 46，47)* | *Hexon* |
| *Human* *Alphaherpesvirus 1(Herpes Simplex Virus 1)* | *UL30* |
| *Human Alphaherpesvirus 2(Herpes Simplex Virus 2)* | *UL30* |
| *Human Alphaherpesvirus 3（Varicella Zoster Virus）* | *ORF38* |
| *Human Gammaherpesvirus 4（Epstein-Barr Virus）* | *late membrane protein1* |
| *Human Betaherpesvirus 5（Cytomegalovirus）* | *UL123* |
| *Human Betaherpesvirus 6（RoseolaVirus 6）* | *U31* |
| *Human Betaherpesvirus 7（RoseolaVirus 7）* | *U57* |
| *Bocaparvovirus primate 1、2、3、4* | *NP1、VP1-VP2* |
| *Human Rhinovirus* | *5’UTR、VP4-VP2* |
| *Human Rhinovirus A（A1-2、A7-13、A15-16、A18-25、A28-34、A36、A38-41、A43、A45-47、A49-51、A53-68、A71、A73-78、A80-82、A85、A88-90、A94-96、A98、100-101、103、105-106）* | *5’UTR、VP4-VP2* |
| *Human Rhinovirus B（B3-6、B14、B17、B26、B27、B35、B37、B42、B48、B52、B69、B70、B72、B79、B83、B84、B86、B91-93、B97、B99）* | *5’UTR、VP4-VP2* |
| *Human Rhinovirus C（C1-3、C5-9、C11-13、C15、C17-19、C22-24、C26、C30-33、C35、C40-44、C47、C53、C55）* | *5’UTR、VP4-VP2* |
| *Human Respiratory Syncytial Virus-A* | *fusion glycoprotein* |
| *Human Respiratory Syncytial Virus-B* | *fusion glycoprotein* |
| *Human Parainfluenza Virus 1* | *HN glycoprotein* |
| *Human Parainfluenza Virus 3* | *nucleocapsid protein gene* |
| *Human Parainfluenza Virus 2* | *nucleocapsid protein gene* |
| *Human Parainfluenza Virus 4* | *phosphoprotein gene* |
| *Mumps Virus* | *NP、SH* |
| *Influenza A Virus** | *HA/NA* |
| *Influenza B Virus* | *Seg8* |
| *Human Parvovirus B19* | *VP2* |
| *Coxsackievirus( A1-A3、A5-A8、A10、A12-A22、B1、B3-B6)* | *VP1* |
| *Echovirus ( E1、E3、E4-E7、E9、E11、E25、E30、E32)* | *VP1* |
| *Enterovirus (A71、A90、A92、A120、A123、B69、C99、C105、C109、C113、**C116、D68、D94、D111、J103)* | *VP1* |
| *Human Metapneumovirus* | *nucleocapsid protein gene* |
| *Measles Virus Genotype* | *nucleocapsid protein gene* |
| *Rubella Virus Genotype* | *nucleocapsid protein gene* |
| *Human Coronavirus-229E* | *nucleocapsid protein gene* |
| *Human Coronavirus-NL63* | *nucleocapsid protein gene* |
| *Human Coronavirus-OC43* | *nucleocapsid protein gene* |
| *Human Coronavirus-HKU1* | *replicase 1b* |
| *Severe acute respiratory syndrome coronavius, SARS-CoV* | *S* |
| *Severe acute respiratory syndrome coronavirus 2,SARS-CoV-2* | *ORF1ab* |
| *Middle East Respiratory Syndrome Coronavirus,MERS* | *orf1ab* |
| *Escherichia coli* | *chuA, yjaA and TspE4.C2* |
| *Staphylococcus aureus* | *nuc* |
| *Klebsiella pneumoniae* | *phoE* |
| *Acinetobacter baumannii* | *OXA-51* |
| *Pseudomonas aeruginosa* | *regA* |
| *Stenotrophomonas maltophilia* | *atpD* |
| *Streptococcus pneumoniae* | *Ply* |
| *streptococcus dysgalactiae* | *recD* |
| *Streptococcus intermadius* | *recD* |
| *Streptococcus anginosus* | *recD* |
| *Streptococcus constellatus* | *cpn60* |
| *Streptococcus pyogenes* | *CsrRS* |
| *Streptococcus agalactiae (group B streptococcus or GBS)* | *cfb* |
| *Peptostreptococcus anaerobius* | *trpE* |
| *Mycobacterium tuberculosis* | *IS6110* |
| *Mycobacterium bovis* | *IS6110* |
| *Mycobacterium africanum* | *IS6110* |
| *Mycobacterium canetti* | *IS6110* |
| *Mycobacterium avium* | *hsp65* |
| *Mycobacterium intracellulare* | *hsp65* |
| *Mycobacterium asiaticum* | *hsp65* |
| *Mycobacterium szulgai* | *hsp65* |
| *Mycobacterium ulcerans* | *hsp65* |
| *Mycobacterium xenopi* | *hsp65* |
| *Mycobacterium scrofulaceum* | *hsp65* |
| *Mycobacterium simiae* | *hsp65* |
| *Mycobacterium novum* | *hsp65* |
| *Mycobacterium haemophilum* | *hsp65* |
| *Mycobacterium botniense* | *hsp65* |
| *Mycobacterium kansasii* | *hsp65* |
| *Mycobacterium abscessus* | *hsp65* |
| *Mycobacterium chelonae* | *hsp65* |
| *Mycobacterium lepromatosis* | *hsp65* |
| *Neisseria gonorrhoeae* | *SE147_04855* |
| *Neisseria meningitidis* | *ctrA* |
| *Bacillus anthracis* | *PxO2 gene* |
| *Bacillus cereus* | *rpoB* |
| *Arcanobacterium haemolyticum* | *pld* |
| *Corynebacterium diphtheriae* | *rpoB* |
| *Fusobacterium necrophorum* | *gyrB* |
| *Haemophilus influenzae* | *hpd、fucK* |
| *Haemophilus parainfluenzae* | *yidC* |
| *Nocardia abscessus* | *rpmG* |
| *Nocardia asteroides* | *rpmG* |
| *Nocardia brasiliensis* | *rpmG* |
| *Serratia marcescens* | *luxS* |
| *Moraxella catarrhalis* | *copB* |
| *Bordetella pertussis* | *IS481* |
| *Bordetella parapertussis* | *IS1001* |
| *Bordetella bronchiseptica* | *IS481* |
| *Bordetella holmesii* | *ISL3* |
| *Legionella pneumophila* | *infC* |
| *Elizabethkingia meningoseptica* | *secY* |
| *Coxiella burnetii* | *IS110* |
| *Brucella abortus* | *dnaK* |
| *Brucella anthropi* | *dnaK* |
| *Brucella melitensis* | *dnaK* |
| *Pneumocystis carinii* | *mitochondrion* |
| *Pneumocystis jirovecii* | *beta-tubulin* |
| *Cryptococcus neoformans* | *mitochondrion* |
| *Cryptococcus gattii* | *mitochondrion* |
| *Trichosporon* | *IGS1* |
| *Mucor* | *mitochondrion* |
| *Candida albicans* | *COB* |
| *Candida tropicalis* | *COB* |
| *Candida dubliniensis* | *COB* |
| *Candida parapsilosis* | *COB* |
| *Aspergillus flavus* | *mitochondrion* |
| *Aspergillus fumigatus* | *mitochondrion* |
| *Aspergillus niger* | *mitochondrion* |
| *Aspergillus terreus* | *mitochondrion* |
| *Aspergillus nidulans* | *GAPDH* |
| *Histoplasma capsulatum* | *GAPDH* |
| *Mycoplasmoides pneumoniae* | *mgpA* |
| *Chlamydia pneumoniae* | *ompA* |
| *Chlamydia psittaci* | *OmpA* |
| *Chlamydia trachomatis* | *OmpA* |

*Design probes for full-length regions of 16 HA subtypes and 9 NA subtypes

**SUPPLEMENTARY TABLE 2** Revalidation results of pathogens detected positively exclusively by RP-MT-Capture NGS

| Pathogen | Sample ID | Real-time PCR validation results（CT value） |
| --- | --- | --- |
| SARS-CoV-2 | TAC-1 | / |
|  | TAC-67 | / |
|  | TAC-70 | / |
|  | TAC-80 | 29 |
|  | TAC-85 | / |
|  | TAC-105 | / |
|  | TAC-106 | 34 |
|  | TAC-108 | 36 |
|  | TAC-113 | / |
|  | TAC-123 | 33 |
|  | TAC-133 | 26 |
|  | TAC-142 | 28 |
|  | TAC-155 | / |
| Human betaherpesvirus 6 | TAC-149 | / |
| Influenza B virus | TAC-159 | 30 |

**supplementary table 3** Revalidation results of pathogens detected positively exclusively by TaqMan array

| Pathogen | Sample ID | Real-time PCR Results（CT value） |
| --- | --- | --- |
| Human coronavirus HKU1 | TAC-97 | 34 |
| Mycoplasma pneumoniae | TAC-92 | 33 |

**SUPPLEMENTARY TABLE 4** Comparison of pathogens detected by RP-MT-Capture NGS and mNGS in 38 samples.

| Sample ID | Pathogen | RP-MT-capture NGS  (RPM*) | mNGS (RPM*) | Folds* |
| --- | --- | --- | --- | --- |
| TAC-11 | Influenza A virus H3N2 H | 207859.9 | 153.3 | 1355.6 |
| TAC-31 | Influenza A virus H3N2 H | 187503.9 | 205.9 | 910.6 |
| TAC-144 | Influenza A virus H3N2 H | 130735.9 | 438.1 | 298.4 |
| TAC-129 | Influenza A virus H3N2 H | 10598.5 | 8.7 | 1213.4 |
| TAC-146 | Influenza A virus H3N2 H | 22656.4 | 5.7 | 4005.0 |
| TAC-104 | Influenza A virus H3N2 H | 9565.1 | 6.0 | 1600.0 |
| TAC-138 | Influenza A virus H3N2 H | 977.3 | 1.6 | 622.8 |
| TAC-133 | Influenza A virus H3N2 H | 2040.6 | 0.6 | 3251.4 |
| TAC-113 | Influenza A virus H3N2 H | 653.0 | 0.4 | 1834.7 |
| TAC-122 | rhinovirus A106 | 427882.4 | 114.1 | 3751.0 |
| TAC-135 | Human rhinovirus sp. | 10938.5 | 0.7 | 15901.8 |
| TAC-140 | rhinovirus A7 | 14895.6 | 0.3 | 42738.9 |
| TAC-123 | rhinovirus A106 | 10624.7 | 0.0 | / |
| TAC-121 | Rhinovirus A | 14260.4 | 0.8 | 17668.1 |
| TAC-111 | rhinovirus C1 | 305.8 | 0.0 | / |
| TAC-122 | Human coronavirus 229E | 55918.2 | 114.1 | 3751.0 |
| TAC-145 | Human coronavirus 229E | 496414.0 | 2244.3 | 221.2 |
| TAC-134 | Human coronavirus 229E | 272.7 | 1.7 | 159.4 |
| TAC-41 | Human coronavirus OC43 | 323758.2 | 0.0 | / |
| TAC-117 | Human parainfluenza virus 1 | 25762.0 | 0.0 | / |
| TAC-110 | Human parainfluenza virus 3 | 6992.8 | 0.0 | / |
| TAC-119 | Human parainfluenza virus 4a | 7718.7 | 0.0 | / |
| TAC-149 | Human alphaherpesvirus 1 | 1037.7 | 5.5 | 187.4 |
| TAC-121 | Human alphaherpesvirus 1 | 1292.1 | 0.0 | / |
| TAC-109 | Human gammaherpesvirus 4 | 1954.1 | 9.1 | 213.8 |
| TAC-132 | Human gammaherpesvirus 4 | 139.8 | 1.3 | 104.6 |
| TAC-155 | Stenotrophomonas maltophilia | 21071.4 | 2056.0 | 10.2 |
| TAC-156 | Stenotrophomonas maltophilia | 43085.1 | 10794.0 | 4.0 |
| TAC-158 | Stenotrophomonas maltophilia | 25038.4 | 7992.1 | 3.1 |
| TAC-157 | Stenotrophomonas maltophilia | 558.5 | 352.3 | 1.6 |
| TAC-125 | Stenotrophomonas maltophilia | 203.8 | 126.1 | 1.6 |
| TAC-60 | Bordetella bronchiseptica | 670.2 | 32.2 | 20.8 |
| TAC-84 | Bordetella bronchiseptica | 686.4 | 14.7 | 46.7 |
| TAC-39 | Moraxella catarrhalis | 2470.1 | 1390.1 | 1.8 |
| TAC-80 | Moraxella catarrhalis | 780.2 | 381.5 | 2.0 |
| TAC-48 | Klebsiella pneumoniae subsp. pneumoniae | 604.8 | 454.5 | 1.3 |
| TAC-115 | Streptococcus pyogenes MGAS9429 | 297.9 | 67.2 | 4.4 |
| TAC-13 | Candida tropicalis | 218.4 | 0.0 | / |

*RPM=reads/Total reads×1000000

*Folds=RP-MT-capture NGS (RPM)/mNGS (RPM)

**SUPPLEMENTARY TABLE 5** The full panel of detectable pathogens for TaqMan Respiratory Tract Microbiota Comprehensive Card^a^

| **Target type** | **Nucleic acid type** | **Assay name** | **Target organism** |
| --- | --- | --- | --- |
| Virus | DNA | *AdV_1of2* | *Adenovirus* |
|  |  | *AdV_2of2* | *Adenovirus* |
|  |  | *HBoV* | *Human Bocavirus* |
|  |  | *HHV3* | *Human herpesvirus 3 (HHV3 – Varicella zoster Virus)* |
|  |  | *HHV4* | *Human herpesvirus 4 (HHV4 – Epstein-Barr Virus)* |
|  |  | *HHV5* | *Human herpesvirus 5 (HHV5 – Cytomegalovirus)* |
|  |  | *HHV6* | *Human herpesvirus 6 (HHV6)* |
|  | RNA | *CoV_229E* | *Human Coronavirus 229E* |
|  |  | *CoV_HKU1* | *Human Coronavirus HKU1* |
|  |  | *CoV_NL63* | *Human Coronavirus NL63* |
|  |  | *CoV_OC43* | *Human Coronavirus OC43* |
|  |  | *EV_pan* | *Human Enterovirus (pan assay)* |
|  |  | *EV_D68* | *Human Enterovirus D68* |
|  |  | *hMPV* | *Human Metapneumovirus (hMPV)* |
|  |  | *hPIV1* | *Human Parainfluenza virus 1* |
|  |  | *hPIV2* | *Human Parainfluenza virus 2* |
|  |  | *hPIV3* | *Human Parainfluenza virus 3* |
|  |  | *hPIV4* | *Human Parainfluenza virus 4* |
|  |  | *RSVA* | *Human Respiratory Syncytial Virus A (RSVA)* |
|  |  | *RSVB* | *Human Respiratory Syncytial Virus B (RSVB)* |
|  |  | *RV_1of2* | *Human Rhinovirus 1/2* |
|  |  | *RV_2of2* | *Human Rhinovirus 2/2* |
|  |  | *Flu_A_pan* | *Influenza A* |
|  |  | *Flu_A_H1* | *Influenza A/H1-2009* |
|  |  | *Flu_A_H3* | *Influenza A/H3* |
|  |  | *Flu_B_pan* | *Influenza B* |
|  |  | *Measles* | *Measles virus* |
|  |  | *Mumps* | *Mumps virus* |
|  |  | *JEV* | *Epidemic type B encephalitis virus/Japanese Encephalitis Virus* |
|  |  | *Rubella virus* | *Rubella virus* |
|  |  | *SARS-CoV-2 ORF1ab gene* | *SARS-CoV-2（orf1ab）* |
|  |  | *SARS-CoV-2 N gene* | *SARS-CoV-2（N）* |
|  |  | *SARS-CoV-2 S gene* | *SARS-CoV-2（S）* |
| Bacteria | DNA | *B.pertussis* | *Bordetella pertussis* |
|  |  | *Bordetella* | *Bordetella parapertussis /pertussis* |
|  |  | *H.influenzae* | *Haemophilus influenzae* |
|  |  | *M.pneumoniae* | *Mycoplasma pneumoniae* |
|  |  | *S.pneumoniae* | *Streptococcus pneumoniae* |
|  |  | *C.pneumoniae* | *Chlamydophila pneumoniae* |
|  |  | *B.holmesii* | *Bordetella holmesii* |
|  |  | *K.pneumoniae* | *Klebsiella pneumoniae* |
|  |  | *L.pneumophila* | *Legionella pneumophila* |
|  |  | *M.catarrhalis* | *Moraxella catarrhalis* |
|  |  | *S.aureus* | *Staphylococcus aureus* |
|  |  | *M. tuberculosis* | *Mycobacerium tuberculosis complex* |
|  |  | *N. meningitidis* | *Neisseria meningitidis* |
| Control | RNA | *Production Control* | *Human 18S ribosomal RNA gene* |
|  | DNA | *Aamplification Control* | *Human RNase P RPPH1 gene* |

**^a^** The TaqMan Respiratory Tract Microbiota Comprehensive Card used in this study can detect key respiratory pathogens using real-time fluorescent quantitative PCR technology. This chip includes optimized TaqMan Assays for 42 types of respiratory pathogens, human RNase P gene (RPPH1) control, and human 18S ribosomal RNA gene control (production control).
